# Anion–Cation Co-Doped g-C_3_N_4_ Porous Nanotubes with Efficient Photocatalytic H_2_ Evolution Performance

**DOI:** 10.3390/nano12172929

**Published:** 2022-08-25

**Authors:** Xiaohan Zhang, Tong Li, Chun Hu, Xiutong Yan, Kai Qiao, Zhihong Chen

**Affiliations:** 1 Institute of Environmental Research at Greater Bay, Key Laboratory for Water Quality and Conservation of the Pearl River Delta, Ministry of Education, Guangzhou University, Guangzhou 510006, China; 2 School of Energy and Environmental Engineering, University of Science and Technology Beijing, Beijing 100083, China

**Keywords:** photocatalytic H_2_ evolution, g-C_3_N_4_, anion–cation co-doping, nanotube

## Abstract

Graphitic C_3_N_4_-based materials are promising for photocatalytic H_2_ evolution applications, but they still suffer from low photocatalytic activity due to the insufficient light absorption, unfavorable structure and fast recombination of photogenerated charge. Herein, a novel anion–cation co-doped g-C_3_N_4_ porous nanotube is successfully synthesized using a self-assembly impregnation-assisted polymerization method. Ni ions on the surface of the self-assembly nanorod precursor can not only cooperate with H_3_P gas from the thermal cracking of NaH_2_PO_2_ as an anion–cation co-doping source, but, more importantly, suppress the shape-collapsing effect of the etching of H_3_P gas due to the strong coordinate bonding of Ni-P, which leads to a Ni and P co-doped g-C_3_N_4_ porous nanotube (PNCNT). Ni and P co-doping can build a new intermediate state near the conduction band in the bandgap of the PNCNT, and the porous nanotube structure gives it a higher BET surface area and light reflection path, showing a synergistic ability to broaden the visible-light absorption, facilitate photogenerated charge separation and the light-electron excitation rate of g-C_3_N_4_ and provide more reaction sites for photocatalytic H_2_ evolution reaction. Therefore, as expected, the PNCNT exhibits an excellent photocatalytic H_2_ evolution rate of 240.91 μmol·g^−1^·h^−1^, which is 30.5, 3.8 and 27.8 times as that of the pure g-C_3_N_4_ nanotube (CNT), single Ni-doped g-C_3_N_4_ nanotube (NCNT) and single P-doped g-C_3_N_4_ nanotube (PCNT), respectively. Moreover, the PNCNT shows good stability and long-term photocatalytic H_2_ production activity, which makes it a promising candidate for practical applications.

## 1. Introduction

The crises of resource scarcity and environmental disruption caused by the excessive use of fossil fuels have profoundly affected the sustainable development of human beings. As a well-known clean energy, hydrogen (H_2_) energy is widely regarded as an ideal substitute for fossil fuels due to its high energy density and lack of carbon dioxide emission. In recent decades, obtaining H_2_ from photocatalytic water splitting reaction has received an increasing amount of attention due to its characteristics of clean and low energy consumption. Among the reported visible-light-driven photocatalysts, graphite carbon nitride (g-C_3_N_4_) has attracted increasing attention in the field of photocatalytic H_2_ production [1,2,3] due to its suitable conduction band potential for water reduction reaction, preeminent chemical and thermal stability. However, the photocatalytic activity of pristine bulk g-C_3_N_4_ is greatly restricted by its intrinsically low specific surface area and inadequate visible-light response range, and the rapid recombination of photogeneration electron–hole pairs originates from its organic π conjugated structure [4,5,6,7]. Therefore, a variety of g-C_3_N_4_-based photocatalysts with superior photocatalytic activity have been synthesized through multiple strategies, such as element doping [8,9], heterojunction construction [10,11,12,13] and morphology regulation [14,15,16].

Morphological control through constructing 3D structures, especially hollow tubular structures, has obtained extensive attention in developing high-efficiency g-C_3_N_4_-based photocatalysts through element doping. Compared with the traditional bulk g-C_3_N_4_, the hollow g-C_3_N_4_ nanotube structure can enhance the light absorption ability through diffuse reflection and promote the migration of photogenerated carriers along the one-dimensional path, which offers a faster electron transfer channel and is beneficial to the recovery needed for practical applications [17]. For instance, Chen et al. [18] converted 2D g-C_3_N_4_ nanosheets into 1D nanotubes through the induction of phosphine gas. The formation of the nanotubes increased the S_BET_ of the photocatalyst and created a porous surface. However, this method is complicated and has a long preparation period. Zhang et al. [19] provided a simple thermal polymerization synthesis method to build g-C_3_N_4_ nanotubes by adding a mixture of melamine and cyanic acid in a fixed proportion to form a supramolecular precursor, but this strategy requires the use of both raw materials, which is often limited by the economy in the practical application process. Li et al. [20] further simplified the preparation process and directly prepared g-C_3_N_4_ nanotubes using the in situ transformation of melamine and molecular self-assembly to form supramolecular intermediates. It is worth noting that the structure of ordered intermediates is formed through the strong saturation from hydrogen bonds in the process of self-assembly, resulting in the g-C_3_N_4_ nanotubes with specific morphologies, which is very important to enhance the ability of photocatalytic reactions. The prepared porous nanotube structure broadens light absorption and facilitates the transfer of the photocarrier along the one-dimensional structure of nanotubes, resulting in higher photogenerated carrier separation efficiency.

In addition, various existing researches have proven that implanting the anion–cation into the g-C_3_N_4_ frameworks could regulate their bandgap structure for broader visible-light absorption, facilitate the bulk phase and interface charge separation and lead to enhanced photocatalytic activity for H_2_ evolution [21,22,23]. For instance, Qiao et al. [24] found that a new intermediate energy level was formed in the bandgap of g-C_3_N_4_ via the insertion of P into its skeleton, which received the electrons generated by light excitation from VB and thus improved the separation of photocarriers. Fang et al. [25] demonstrated that the insertion of Fe(III) into the framework of g-C_3_N_4_ through impregnation, which enhanced the sensitivity of the catalyst to visible light, induced an interfacial charge transfer (IFCT) for faster charge separation and obtained a superior photocatalytic activity. Moreover, relative to the doping of a single element into g-C_3_N_4_, the co-doping of anion–cation has been proven to be a more promising strategy for improving photocatalytic activity, which is ascribed to its synergistic effect in modifying the electronic structure, reducing defect positions, accelerating charge transfer and broadening the visible light absorption of g-C_3_N_4_ [26,27,28]. To date, various g-C_3_N_4_-based photocatalysts with different combinations of anion–cation co-doping have been developed and show obvious enhanced photocatalytic activity in comparison to the doping of a single element. For example, Xu et al. [29] reported a P/K-co-doped porous g-C_3_N_4_ prepared by one-step thermal polymerization with K_2_HPO_4_ as a double-functional dopant, which showed 2.8 times more photocatalytic activation than the pristine g-C_3_N_4_. The improvement of photocatalytic activity is attributed to the narrowed bandgap, which allows for more efficient use of visible light and faster mass/charge transfer. In addition, Na-O [30], Fe-P [31], K-I [32], Na-S [33], Co-O [34] and other co-doping systems have also been successfully incorporated into g-C_3_N_4_ to improve its photocatalytic performance.

More recently, the combination of element doping and nanotube control has been demonstrated to be a more efficient way to improve photocatalytic activity and benefit the light absorbance of g-C_3_N_4_. For example, our previous research reported g-C_3_N_4_ nanotubes with carbon-ring doping prepared through the self-assembly assisted thermal polymerization method, which showed superior photocatalytic H_2_ production performance with outstanding recycling stability [35]. He et al. [36] prepared a hierarchical g-C_3_N_4_ tube with oxygen doping and carbon defects through the self-templating method, which achieved satisfactory recycling photocatalytic H_2_ evolution due to the larger surface area, light scattering, the shorter charge transfer distance and the creation of midgap states. Moreover, Sun et al. [37] found that the photocatalytic H_2_ evolution ability of g-C_3_N_4_ was significantly enhanced through the modification of anion doping and tube formation. The P/S co-doped g-C_3_N_4_ effectively expanded light absorption; thus, more charge carriers were excited in order to be distributed in the hydrogen production process. Cabot et al. [38] successfully prepared Ni/O co-doped nanotubes, which promoted the rapid separation of charge carriers in the ORR process and improved the ability of catalysts to produce hydrogen peroxide under light irradiation. Inspired by these results, it can be speculated that g-C_3_N_4_ nanotubes with anion–cation doping will combine the advantages of the above-mentioned co-doping and tubular structure and result in a high-efficiency g-C_3_N_4_ photocatalyst for H_2_ production, but this is still rarely reported.

Herein, an anion–cation co-doped g-C_3_N_4_ nanotube (PNCNT) was successfully prepared through self-assembly impregnation-assisted thermal polymerization method. The crystalline phase, morphology features and surface states of the PNCNT were characterized, and the results revealed that the P and Ni atoms were co-doped into the g-C_3_N_4_ framework, and the Ni atom could prevent the cracking of hollow tubular structures. The optical, photochemical, luminescent and electronic distribution properties were measured, and it was found that P/Ni-co-doped and porous hollow tubular structures can synergistically enhance photogenerated carrier transport, light absorption and surface catalytic reactions. Therefore, the PNCNT showed a photocatalytic H_2_ evolution efficiency of up to 954.5 μmol/g after irradiation for 4 h under visible light, which is approximately 29.1 times as that of a CNT. This work enriches and expands the idea of developing high-performance g-C_3_N_4_-based photocatalysts through anion–cation co-doping along with morphological control.

## 2. Experiments

### 2.1. Synthesis of Self-Assembled Nanorod Precursor

An amount of 2 g melamine was added into 120 mL deionized water with strong stirring for dissolution in a water bath at 85 °C. The dissolved solution was transferred into a Teflon-lined autoclave and reacted at 180 °C for 8 h. The obtained reaction products were preserved as precursors after washing with deionized water and anhydrous ethanol several times before drying in an oven.

### 2.2. Synthesis of g-C_3_N_4_ Nanotubes (CNTs)

The precursors were loaded into a porcelain crucible and calcined to 500 °C in a tube furnace under a nitrogen atmosphere, and the heating rate was maintained at 2.5 °C/min. The obtained products were ground into fine powders in an agate mortar, denoted as CNTs.

### 2.3. Synthesis of Single P-Doped g-C_3_N_4_ Nanotubes (PCNT), Single Ni-Doped g-C_3_N_4_ Nanotubes (NCNT) and P/Ni Co-Doped g-C_3_N_4_ Nanotubes (PNCNT-x)

First, the precursors were added into the NiCl_2_·6H_2_O solution under strong stirring for 2 h in order to make them homogeneous. The solution mixture was separated through high-speed centrifugal force, and the precipitated material was dried at 60 °C. Second, the composite mixed with NaH_2_PO_2_ was calcinated under the same condition with a CNT. The final resultant sample was named PNCNT-x (x = 5, 10, 15), where x represents 0.5, 1 and 1.5 wt% Ni added to the solution. In addition, the same procedure was used to synthesize the single P-doped g-C_3_N_4_ nanotubes (PCNT) and the single Ni-doped g-C_3_N_4_ nanotubes (NCNT) without adding the NiCl_2_·6H_2_O solution or NaH_2_PO_2_, respectively, before calcination to clarify the effect of single P-doped and single Ni-doped load on the performance of the photocatalyst.

### 2.4. Characterization

The morphology and composition of the catalyst were analyzed using a scanning electron microscope (SEM) equipped with an EDX detector (for the neatness of the manuscript, PNCNT-10 samples were selected to represent PNCNT in the following characterization). Transmission electron microscopy (TEM) was carried out to further observe the surface microstructure. The crystalline structure was measured with an X-ray diffraction (XRD) with a wide-angle scanning range with Cu Kα radiation. The N_2_ adsorption/desorption analysis was conducted by Micromeritics ASAP 2460 specific surface area analyzer. FTIR spectra were measured to reflect the structural information of the catalyst by a Thermo spectrometer, through setting the scanning range from 4000 cm^−1^ to 500 cm^−1^ for a total of 36 scans. X-ray photoelectron spectroscopy (XPS) was obtained on a Thermo Nexsa photoelectron spectrometer equipped with an Al Kα radiation source. The photoluminescence (PL) spectra and the time-resolved PL spectra of the catalyst were recorded to evaluate the transfer and recombination of the photocarriers with a FLS100 fluorescence lifetime spectrophotometer. UV-vis diffuse reflectance spectra (DRS) were used to analyze the light absorption capacity of the catalyst.

### 2.5. Photocatalytic H_2_ Evolution Activity

Photocatalytic H_2_ evolution measurements were accomplished in the Labsolar-6A system (Perfectlight, Beijing). A 300 W Xenon lamp (PLS-SXE300, Perfectlight, Beijing) equipped with a cutoff filter (λ ≥ 400 nm) was used as the light source. Typically, a quartz vessel contained 50mg catalyst and 100 mL aqueous solution with 10 vol.% triethanolamine (TEOA) as a scavenger to quench photo-induced holes was prepared for a photocatalytic reaction. Additionally, 1 wt% Pt was loaded onto the surface of the catalyst through the conventional photo-reduction method. Before every reaction, the optical power meter was used to calibrate the same light intensity, control the reaction temperature at 5 °C through the cooling water circulation system and bubble nitrogen in the system for 30 min to ensure that the reaction system was maintained under anaerobic conditions. During the reaction, the gaseous product was sampled per hour through a rubber plug and detected using gas chromatography (Techcomp GC7900), which was equipped with a TCD detector with N_2_ as the carrier gas.

To evaluate the apparent quantum efficiency (*AQE*) of the photocatalyst, a PCX-50C (Perfectlight, Beijing, China) system equipped with a 300 W Xe-lamp and a 400 nm band pass filter was used to perform photocatalytic H_2_ production under monochromatic light. The *AQE* for H_2_ evolution was calculated using the following equation:

(1) $$\mathrm{AQE}=\frac{number\;of\;reacted\;electrons}{number\;of\;incident\;photons}\times 100\%$$

### 2.6. Photoelectrochemical Measure Experiments

The transient photocurrent time (I-t) and electrochemical impedance spectra (EIS) of the catalyst were investigated using the CHI-730E electrochemical workstation. In an electrochemical workstation with a typical three-electrode system, an Ag/AgCl electrode was used as a reference electrode, the catalyst loaded onto the fluorine-doped tin oxide (FTO) glass as the working electrode and a Pt plate as the counter electrode, respectively. Moreover, the Xe-lamp with a cutoff filter (λ > 400 nm) was utilized as the light source and 0.1 M Na_2_SO_4_ aqueous solution as the electrolyte.

The working electrode preparation process was conducted according to the following steps: 5 mg of photocatalyst was dispersed in a 410 μL mixed solution, consisting of 10 μL of Nafion solution, 300 μL of water and 100 μL of isopropanol under ultrasonic treatment for 60 min. Then, 20 μL of the resultant catalyst solution was spread onto the FTO glass with a fixed area and dried on a heating plate.

## 3. Results

### 3.1. Photocatalyst Structure

**Figure 1:**
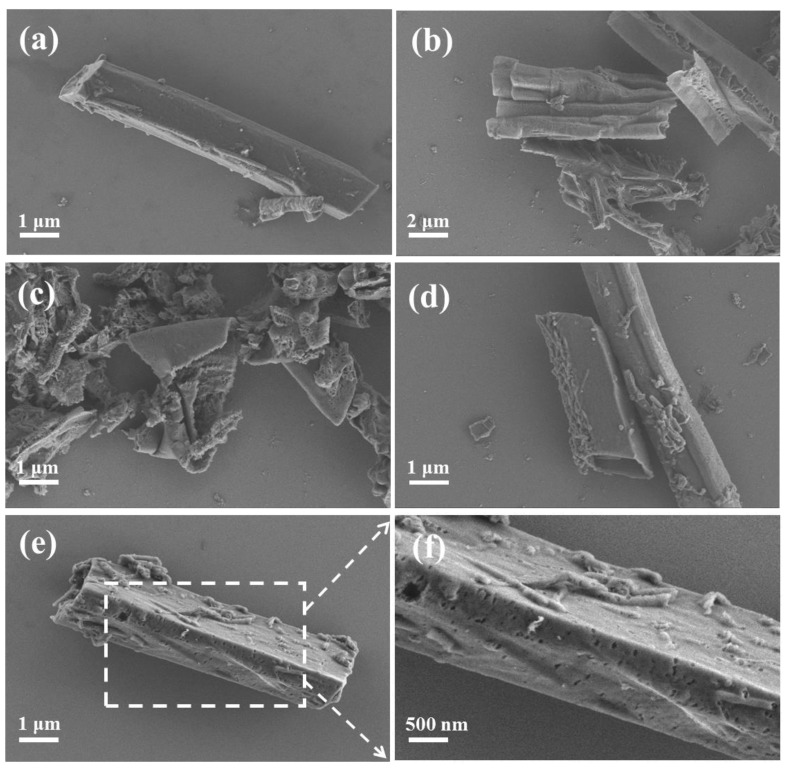
The SEM images of precursor (**a**), CNT (**b**), PCNT (**c**), NCNT (**d**), PNCNT (**e**) and the surface situation of PNCNT samples (**f**).

**Figure 2:**
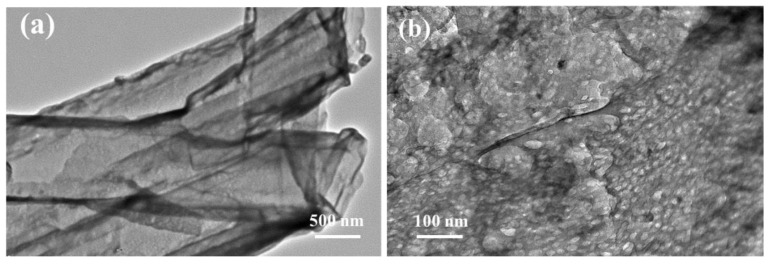
TEM images of hollow tubular morphology (**a**) and surface porous state (**b**) of PNCNT samples.

The morphology and microstructure of the as-prepared samples were investigated using SEM and TEM. As shown in Figure 1, the nanorod precursor prepared through the hydrothermal self-assembly of melamine presented an average length of 3–6 μm. The nanorod precursor was turned into a collapsed nanotube structure after thermal polymerization in a N_2_ atmosphere (CNT), which was due to the weak binding force of hydrogen bonds. With the addition of NaH_2_PO_2_ into the thermal polymerization process, the nanorod precursor was completely broken and converted into fragmentized sheets (PCNT) due to the etching effect of H_3_P gas from the cracking of NaH_2_PO_2_. After the impregnation of the Ni ion into the nanorod precursor, both the NCNT and PNCNT showed a clear and intact nanotube morphology, suggesting that the Ni ion could suppress the shape collapsing and destruction of the nanorod precursor under the thermal polymerization process, which might be attributed to the formation of Ni-P and strong coordinate bonding between Ni and melamine. Additionally, compared with NCNT, an amount of mesopores were formed on the tube wall of PNCNT, which could offer more reaction sites for photocatalytic H_2_ reaction (Figure 1f). As shown in the EDX spectroscopy of PNCNT (Figure S1), Ni and P were highly dispersed on the surface of the nanotube, demonstrating the co-doping of Ni and P on the surface of PNCNT. The TEM images (Figure 2) of PNCNT clearly reveal the hollow tubular structure of PNCNT with abundant nanopore for surface photocatalytic H_2_ evolution reaction.

**Figure 3:**
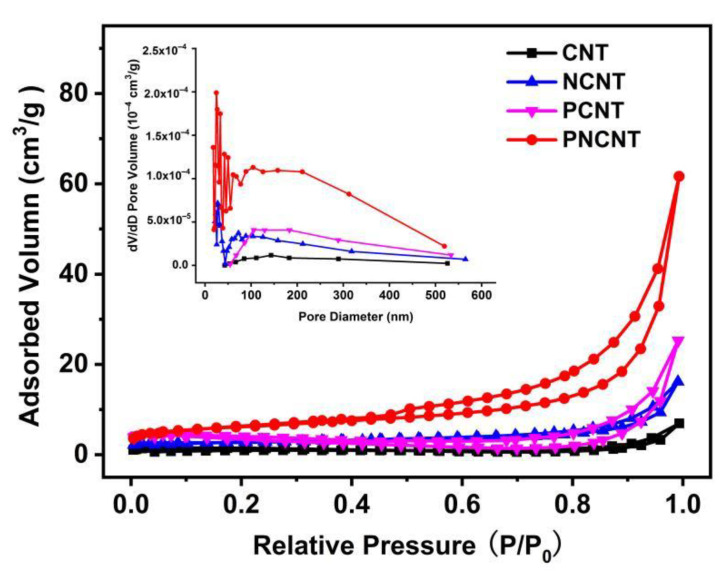
Nitrogen adsorption–desorption isotherms (inset shows the pore size distribution curves) of CNT, NCNT, PCNT and PNCNT samples.

To further study the porous hollow tubular structure of the PNCNT, the N_2_ adsorption–desorption isotherms of the CNT, NCNT, PCNT and PNCNT samples were measured (Figure 3). Table S1 lists the corresponding Brunauer–Emmett–Teller (BET) surface areas, pore volumes and pore diameters. It is clear that all the as-prepared samples displayed type IV isotherms with H_3_ hysteresis loops, indicating the mesoporous characteristic pore structure of the as-prepared g-C_3_N_4_-based materials. The BET surface area of the CNT was 4.115 m^2^/g, which showed a slight enhancement to 8.763 m^2^/g and 8.748 m^2^/g with single Ni-doping and P-doping, respectively, implying that single Ni-doping or P-doping is highly beneficial to enhance the surface area of the CNT, but the effect is not significant. In contrast, with the co-doping of Ni and P, the BET surface of the PNCNT rapidly increased to 22.257 m^2^/g, which is much larger than that of the PCNT and NCNT, suggesting a synergistic effect of building pores on the surface of the CNT tubular structure. The pore size distribution of the PCNT and NCNT was concentrated in the range of 50–300 nm, while the PNCNT showed a clearly additional pore size distribution at 1–50 nm, which is consistent with the SEM and TEM results and demonstrates that the synergistic effect of Ni and P co-doping has an excellent nanopore-producing ability. The additional pore size distribution might be ascribed to the ammonia gas emission along with H_3_P etching in the calcination process. The nanoporous structure of the g-C_3_N_4_ nanotube can increase the activity site number and light reflection path for the photocatalytic H_2_ evolution reaction.

**Figure 4:**
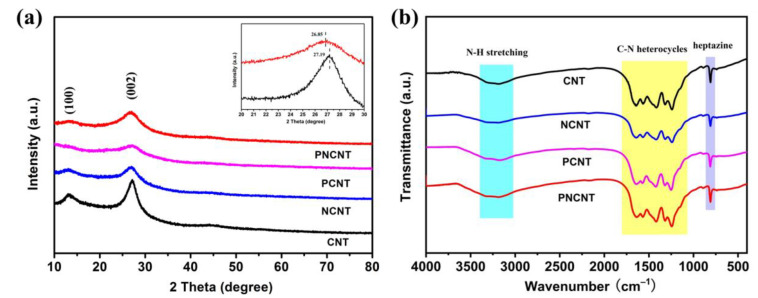
XRD pattern (**a**) and FTIR spectra (**b**) of CNT, NCNT, PCNT and PNCNT samples.

The crystalline and chemical composition of the CNT, PCNT, NCNT and PNCNT was investigated using XRD and FTIR measurements. As shown in Figure 4a, CNT and NCNT maintained the typical phase structure of g-C_3_N_4_ with characteristic diffraction peaks at 13.4 and 27.5°, which is related to the (100) inter-planar graphitic stacking and the (002) inter-planar stacking peak, indicating that neither the single Ni doping nor the P/Ni co-doping process destroyed the main crystalline structure of the g-C_3_N_4_ nanotubes. However, the XRD spectra of the PCNT exhibited a significant decrease in the intensity of both the diffraction peaks of the (002) and (100) planes, which suggests that the inter-planar structure of the g-C_3_N_4_ was changed or destroyed due to the H_3_P etching originating from the cracking of NaH_2_PO_2_ in the thermal polymerization process.

On the contrary, with the impregnation of the Ni ion in the nanotube precursor, the PNCNT also maintained the typical characteristic diffraction phase of g-C_3_N_4_, which might test the theory that the addition of Ni is beneficial to reduce the structure etching from H_3_P in order to maintain the nanotube structure. As depicted in Figure 4b, the CNT, PCNT, NCNT and PNCNT revealed almost identical FTIR spectra; a group of strong vibration peaks at 810, 1200–1700 and 3200–3400 cm^−1^ were classified as the typical heterocycle skeletal vibration of g-C_3_N_4_, indicating that the single or co-doping process does not break the main chemical skeleton of g-C_3_N_4_. In addition, the peaks located at 810 cm^−1^ and 1200–1700 cm^−1^ responded to the breathing vibration of the heptazine units of the g-C_3_N_4_ and -CN heterocycles, respectively. Additionally, the wide peak located at 3200–3400 cm^−1^ represented the hydroxyl of the surface-absorbed water and uncondensed amino groups (-NHx).

**Figure 5:**
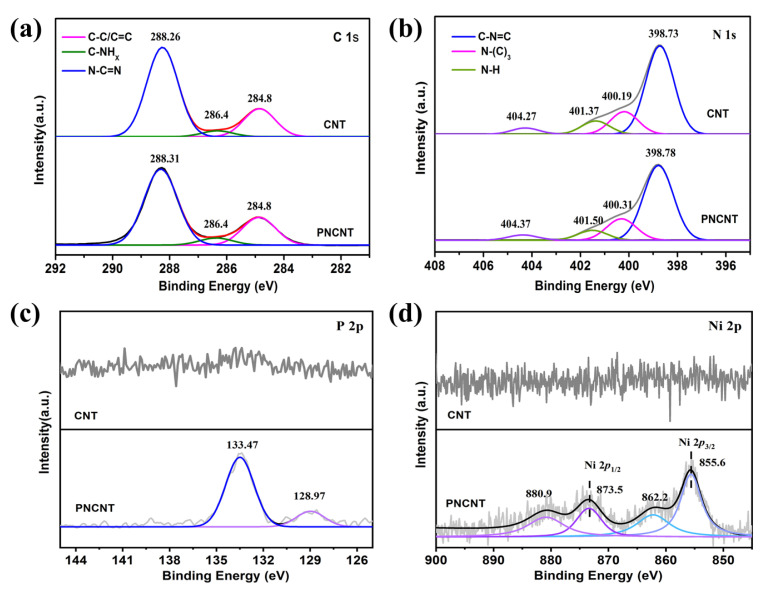
The high-resolution C 1s (**a**), N 1s (**b**), P 2p (**c**) and Ni 2p (**d**) XPS spectra of CNT and PNCNT samples.

**Scheme 1:**
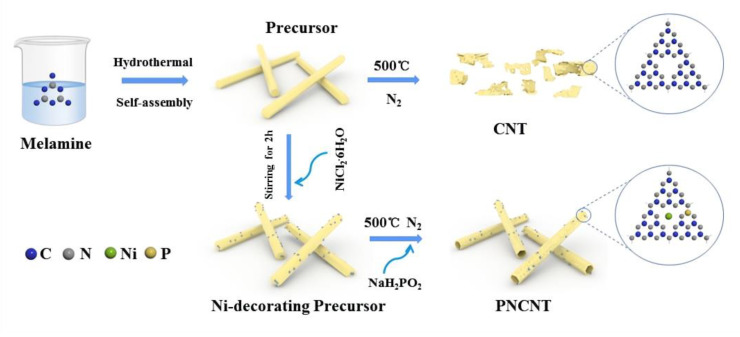
The synthesis process of the PNCNT samples.

XPS measurement was carried out to investigate the surface chemical states of the as-prepared sample and confirm the co-doping of Ni/P into the g-C_3_N_4_ framework. The results are displayed in Figure 5. It is apparent that Figure S2 depicts the photoelectron peaks of C, N and O existing in both the XPS survey spectra of the CNT and PNCNT. Except for C, N and O, the signal related to P and Ni elements can be found in the PNCNT XPS survey spectra, indicating the co-doping of P and Ni into the framework of the g-C_3_N_4_ nanotube. Regarding the C 1s high-resolution spectra (Figure 5a), the characteristic peaks at approximately 284.8, 286.4 and 288.3 eV corresponded to the sp^2^ C-C/C=C, C-NHx bond and sp^2^-bonded carbon (N-C=N), respectively. The N 1s high-resolution spectra (Figure 5b) were separated into four peaks centered at binding energies of 398.7, 400.1, 401.3 and 404.3 eV, corresponding to N-C=N, N-(C)_3_, N-Hx and π-excitation, respectively. It is worth noting that all the peaks of N 1s in the PNCNT displayed a weak positive shift in comparison to the CNT, which was caused by the strong interfacial interactions from the surface electron transfer from the g-C_3_N_4_ to the Ni atom. In the P 2p high-resolution spectra of the PNCNT, two peaks located at 28.97 and 133.47 eV were clearly observed, which is considered to be the P^δ-^ and typical P-N coordination. This implies that the P atom replaces the C atom rather than the N atom in the g-C_3_N_4_ framework because the binding energy of the P-N band is 1–2 eV higher than that of P-C [39]. Two characteristic peaks with satellite peaks of Ni 2p^3/2^ (855.6 eV) and Ni 2p^1/2^ (873.5 eV) were clearly observed in the high-resolution Ni 2p XPS spectra. The peak located at 855.6 eV is assigned to Ni^δ+^, which can be bonded with P^δ-^ to easily form a P–Ni bond to induce electronic interactions. Meanwhile, the peak located at 873.5 eV is assigned to the coordination bond of Ni–N in the triangular hole in the plane of the g-C_3_N_4_ aromatic ring. Based on the above results and discussion, a potential structure of the PNCNT is proposed and shown in Scheme 1.

### 3.2. Photocatalytic Activity of H_2_ Evolution

**Figure 6:**
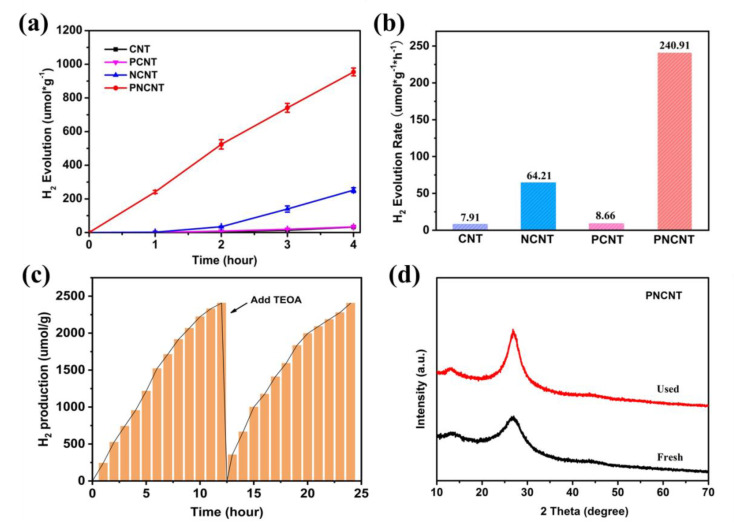
Time courses of photocatalytic H_2_ evolution (**a**) and H_2_ evolution rate (**b**) of CNT, NCNT, PCNT, PNCNT samples under visible light; photocatalytic H_2_ evolution stability test of PNCNT (**c**), the XRD pattern of fresh and used PNCNT after photocatalytic H_2_ evolution stability tests (**d**).

To assess the activity of the as-prepared samples, 10 vol.% TEOA was used as the sacrificial agent in aqueous solution in a photocatalytic H_2_ evolution system with visible-light irradiation, and the results are shown in Figure 6a. Clearly, the CNT showed an extremely low H_2_ production of 32.78 μmol/g under a four-hour irradiation (Figure 6a), which is due to its inadequate visible-light response and severe charge recombination. After single P-doping to the framework of g-C_3_N_4_, the PCNT showed only a negligible improvement in photocatalytic H_2_ production to 33.65 μmol/g, indicating that high-charge recombination from the damage of the nanotube and crystalline structure is responsible for limiting its activity. Comparatively, the photocatalytic H_2_ production of the NCNT increased to 252.2 μmol/g due to the catalytic effect of the Ni atom and nanotube, which induced fast charge separation. The PNCNT exhibited a superior photocatalytic H_2_ production up to 954.5 μmol/g, which was approximately 29.1, 28.4 and 3.8 times as that of the CNT, PCNT and NCNT, respectively. Moreover, the photocatalytic H_2_ production of the PNCNT was higher than the sum of the PCNT and NCNT under the same illumination time. These results confirm that P/Ni co-doping is an effective strategy to enhance the photocatalytic H_2_ evolution activity of the CNT. The synergistic effect of anion–cation co-doping plays a more important role in improving the photocatalytic activity than two single-doping processes. However, an excess amount of Ni doping leads to a decrease in photocatalytic H_2_ production concentration (594.51 μmol/g), which may result from excessive Ni acting as a recombination site on the g-C_3_N_4_ nanotube surface (Figure S3). Additionally, the increasing trend of the hydrogen concentration produced by the catalyst under illumination with time conformed to the pseudo-first-order kinetic equation and the fitted k value showed a consistent result with photocatalytic H_2_ production (Figure 6b). In addition, the apparent quantum efficiency (*AQE*) of the PNCNT was calculated to be 1.66% at 400 nm. Notably, the PNCNT exhibited remarkable stability in the photocatalytic H_2_ production reaction (Figure 6c). The photocatalytic H_2_ evolution rate of the PNCNT gradually decreased during an 8 h reaction due to the consumption of TEOA. With the re-addition of TEOA, the PNCNT recovered to the same photocatalytic H_2_ evolution rates, which proved the good photo-stability and reusability of the PNCNT. Additionally, the PNCNT was collected after the cycle experiment, and the crystal structure stability was assessed through XRD measurement (Figure 6d). It is clear that there was no obvious change in the XRD pattern of the PNCNT before and after the cycle test, further demonstrating the superior stability of the PNCNT under a photocatalytic H_2_ production reaction. Hence, PNCNT, as a well-performing photocatalyst, showed unique advantages in H_2_ evolution compared with other g-C_3_N_4_-based compositions (Table S3).

### 3.3. The Optical Properties and Electrochemical Analysis

**Figure 7:**
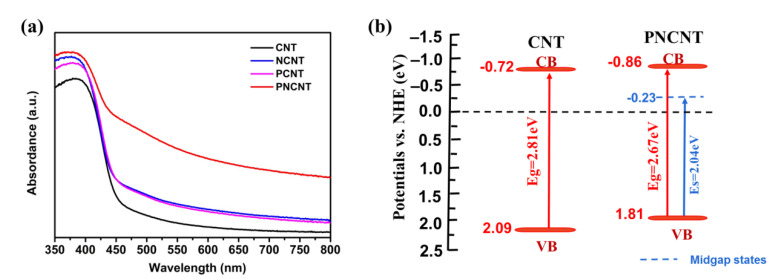
UV-vis diffuse reflection spectra of CNT, NCNT, PCNT and PNCNT samples (**a**) and the band structure of CNT and PNCNT samples (**b**).

The optical properties of photocatalysts were studied using UV-Vis diffuse reflectance spectra. As shown in Figure 7a, the CNT reached an absorption of ca. 450 nm, which corresponded to a band-to-band excitation and an intrinsic bandgap of g-C_3_N_4_. Compared with the CNT, both the PCNT and NCNT showed a trailing absorption along with the intrinsic bandgap, which is related to the formation of P-mid-states and the Ni-induced interfacial charge transfer effect, respectively. Compared with the PCNT and NCNT, the PNCNT showed a mild red shift of the intrinsic bandgap and an obvious trailing absorption in the whole visible-light range, demonstrating that there is a synergistic effect between P and Ni co-doping that vastly extends the visible-light absorption ability of the CNT, which might result from the connection of mid-states and the interfacial charge transfer effect forming a new Urbach tail for sub-bandgap (*Es*). To study the band structure of the PNCNT, the intrinsic bandgap value was calculated using the Tauc equation. Meanwhile, the sub-bandgap value of the PNCNT was estimated using the plots of *(αhν)^1/2^* vs. the energy of the absorbed light due to the indirect bandgap semiconductor property of g-C_3_N_4_, and the results are shown in Figure S4a. The CNT showed only an intrinsic bandgap of 2.81 eV, while the PNCNT displayed not only a narrowed bandgap of 2.67 eV but also a sub-bandgap of 2.04 eV.

The VB positions of the CNT and PNCNT were determined to be 2.09 and 1.81 V vs. NHE, respectively, using XPS valence band spectra. Then, the CB potentials of the samples were obtained according to the following equation:

*E_CB_* = *E_VB_* − *Eg* (2)

**Figure 8:**
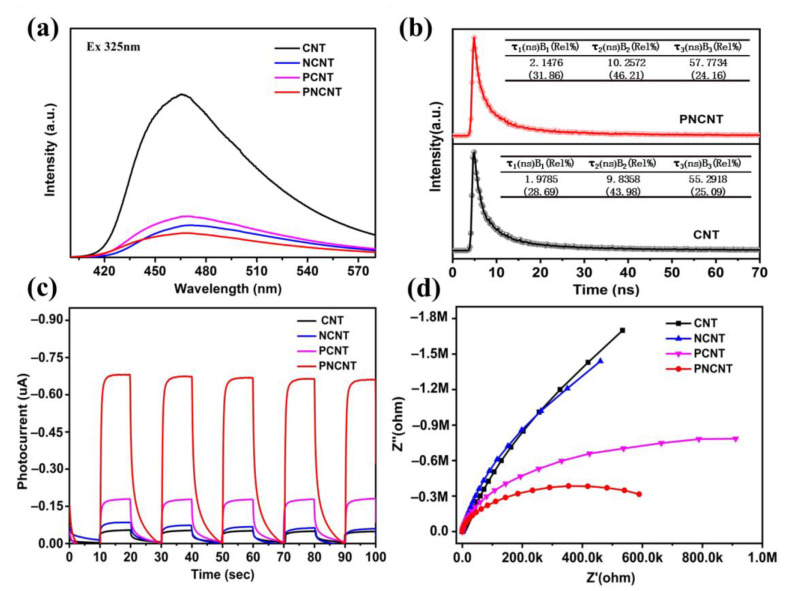
Photoluminescence spectra (**a**), PE-TA spectra of CNT and PNCNT (**b**), the periodic on/off photocurrent response under visible-light irradiation (**c**) and electrochemical impedance spectroscopy plots (**d**) of CNT, NCNT, PCNT and PNCNT samples.

The CB potentials of the CNT and PNCNT were calculated to be −0.72 eV and −0.86 eV. Based on previous research, P-doping and Ni-induced IFCT were near and below the CB position, so the mid-state potentials of the PNCNT were determined to be −0.23 V vs. NHE, which is more negative than the potential of the H^+^/H_2_ reaction, indicating that the electrons on the mid-states are able to evolve H_2_ in thermodynamics. Overall, the mid-states broadened the visible-light absorption and accelerated the transmission of the photo-induced holes from VB to the mid-gap states simultaneously. In order to further investigate the light response of the synergistic effect between P/Ni co-doping, the PL and TRPL spectra and photochemical spectroscopy were applied to study the photogenerated charge transfer behavior. Figure 8a displays the steady-state PL spectra of the CNT, PCNT, NCNT and PNCNT, and the excitation wavelength was set to 325 nm. All the prepared samples showed a broad emission PL peak centered at 470 nm, which is related to the direct electron–hole recombination of intrinsic band-to-band transition. The PL emission peak position was a little higher than the intrinsic bandgap value of g-C_3_N_4_, which is due to the Stokes shift of photoelectric conversion. The peak intensity of the PCNT and NCNT showed an obvious decrease after single doping with P or Ni and showed a sharp quenching with P/Ni co-doping (PNCNT), proving that the charge recombination is inhibited to a greater extent by the co-doping method. Furthermore, the charge separation dynamics of the CNT and PNCNT was investigated using time-resolved PL spectra, and the results are shown in Figure 8b. The lifetime of the photocarriers was fitted using triple-exponential fitting equations, and the average emission lifetimes (τA) of the CNT and PNCNT were calculated to be 18.766 ns and 19.382 ns, respectively, indicating that co-doping benefits the improvement of charge separation efficiency. In addition, compared with single P-doping or Ni-doping, the PNCNT with the P/Ni co-doping showed a much higher photocurrent and smaller arc radius than that of the PCNT and NCNT (Figure 8c,d), demonstrating that co-doping is a more effective route to facilitate photoelectric conversion and interfacial charge transfer, which further confirms the synergistic effect for accelerating charge separation.

**Figure 9:**
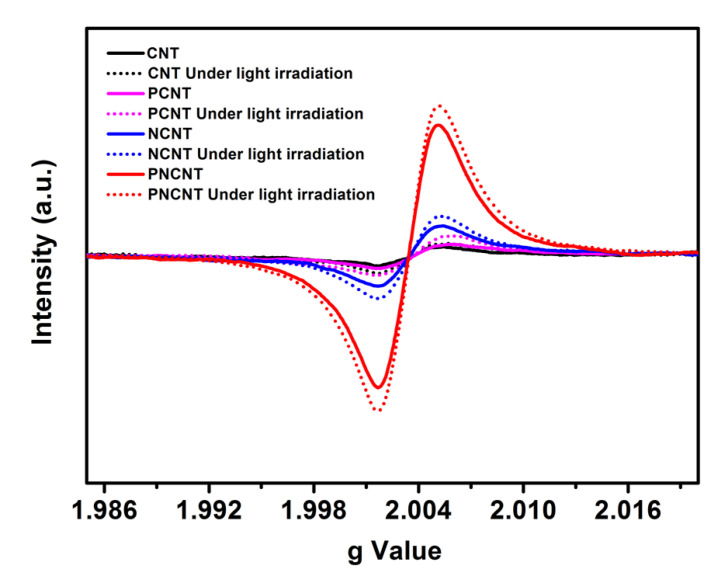
EPR pattern in dark and light irradiation conditions of CNT, NCNT, PCNT and PNCNT samples.

The single-electron state of the CNT, PCNT, NCNT and PNCNT was characterized by electron paramagnetic resonance (EPR) spectra to determine the effect of single and co-doping on the unpaired electrons of heptazine π-conjugated rings. As shown in Figure 9, a single Lorentzian line centered at g = 2.0034 could be clearly observed for all the samples, which corresponded to the unpaired electron on the sp^2^-carbon atoms of the heptazine rings. The PNCNT displayed a significant enhancement in the EPR signal in comparison to the CNT, PCNT and NCNT, suggesting the synergistic effect for the further delocalization of the heptazine π-conjugated rings. More importantly, it was observed that the PNCNT, as expected, still exhibited an increased and highest signal intensity during the irradiation process, suggesting that co-doping is more favorable to the sensitive light response of photo-induced free radical pairs, which promotes the conversion of solar energy to hydrogen energy.

**Scheme 2:**
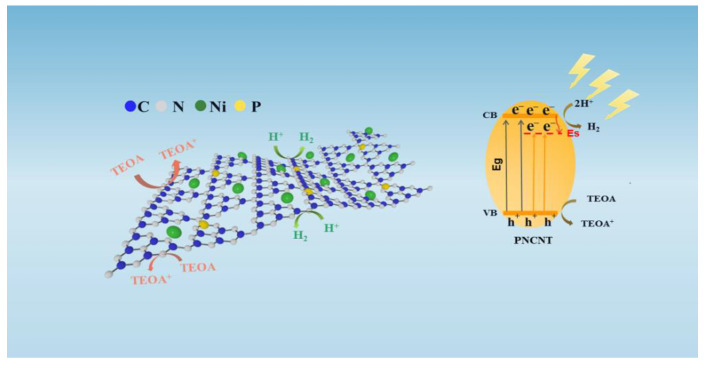
The mechanism of photocatalytic H_2_ evolution on the PNCNT samples.

Therefore, a possible photocatalytic reaction mechanism of the PNCNT for photocatalytic H_2_ evolution was proposed and is illustrated in Scheme 2. The co-doping of P and Ni will create suitable mid-states near and below the CB of the g-C_3_N_4_ nanotube and lead to a sub-bandgap, which can greatly broaden its visible-light absorption. Under visible-light irradiation, the PNCNT is excited by intrinsic and sub-bandgaps to generate electrons on the CB and mid-states and leave holes on the VB. As the potential of mid-states is more positive than the CB, the photogenerated e^−^ is easily transferred from the CB of the PNCNT to the *Es* and reacts with water to produce H_2_.

## 4. Conclusions

In summary, a P/Ni co-doped porous g-C_3_N_4_ nanotube was successfully prepared using the molecular self-assembly impregnation method because Ni-doping can suppress the shape collapse and destruction of the nanotube under the thermal polymerization process through strong coordinate bonding and the formation of Ni-P. P/Ni co-doping can not only enhance visible-light absorption using a sub-bandgap, suppress charge recombination and facilitate photoelectric conversion, but it also creates more pores for photocatalytic reaction sites. As expected, the PNCNT showed an outstanding photocatalytic performance in the H_2_ evolution of 240.91 μmol·g^−1^·h^−1^ under visible light irradiation, which was 30.5 times higher than that of the CNT. Moreover, the PNCNT had good stability in photocatalytic H_2_ evolution when exposed to testing under visible-light irradiation for a long period of time. This work provides a deep insight and a convenient route for constructing high-performance g-C_3_N_4_-based photocatalysts with the combination of surface electronic regulation and nanostructure engineering.

## Data Availability

Not applicable.

## Supplementary Materials

The following supporting information can be downloaded at: https://www.mdpi.com/article/10.3390/nano12172929/s1, Figure S1: The EDX spectrum of PNCNT samples; Figure S2: The XPS survey spectra of CNT and PNCNT samples; Figure S3: Time courses of photocatalytic H_2_ evolution (a) and H_2_ evolution rate (b) of CNT, NCNT, PCNT and PNCNT-x samples under visible light; Figure S4: Plots of *(ahv)^1/2^* vs. photon energy (a) and XPS-VB of CNT and PNCNT (b); Table S1: The BET surface area, pore volume and average pore size of CNT, NCNT, PCNT and PNCNT samples; Table S2: Portion of each peak in the *C 1s* XPS spectra of CNT and PNCNT; Table S3: Comparison with other g-C_3_N_4_-based photocatalysts for HER rate. References [40,41,42,43,44,45] were cited in the Supplementary Materials.
