# Supplementary material for: Anion–Cation Co-Doped g-C3N4 Porous Nanotubes with Efficient Photocatalytic H2 Evolution Performance
Source: Nanomaterials (Basel). 2022 Aug 25;12(17):2929. doi: 10.3390/nano12172929 (PMC9457735; doi:10.3390/nano12172929)
Supplement: Supplementary file 1 [file nanomaterials-12-02929-s001.zip › nanomaterials-1869238-supplementary.pdf]

nanomaterials

Supplementary Materials

Anion–Cation Co-Doped g-C_3N_4 Porous Nanotubes with Efficient Photocatalytic H_2 Evolution Performance

Xiaohan Zhang [1], Tong Li [2], Chun Hu [1], Xiutong Yan [1], Kai Qiao [1] and Zhihong Chen [1,*]

[1] Institute of Environmental Research at Greater Bay, Key Laboratory for Water Quality and Conservation of the Pearl River Delta, Ministry of Education, Guangzhou University, Guangzhou 510006, China
[2] School of Energy and Environmental Engineering, University of Science and Technology Beijing, Beijing 100083, China
* Correspondence: chenzhihong1227@sina.com; Tel.: +86-(020)-39386084

Figure caption

Figure S1. The EDX spectrum of PNCNT samples.

Figure S2. The XPS survey spectra of CNT and PNCNT samples.

Figure S3. Time courses of photocatalytic H_2 evolution (a) and H_2 evolution rate (b) of CNT, NCNT, PCNT and PNCNT-x samples under visible light.

Figure S4. Plots of $(ahv)^{1/2}$ vs. photon energy (a) and XPS-VB of CNT and PNCNT (b).

Table caption

Table S1. The BET surface area, pore volume and average pore size of CNT, NCNT, PCNT and PNCNT samples.

Table S2. Portion of each peak in the C 1s XPS spectra of CNT and PNCNT.

Table S3. Comparison with other g-C_3N_4-baesd photocatalysts for HER rate.

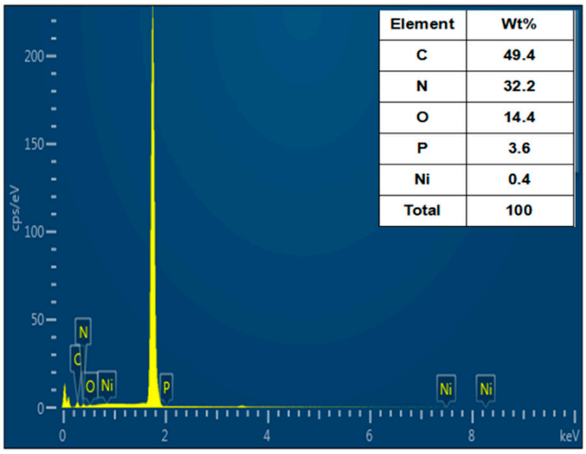

Figure S1. The EDX spectrum of PNCNT samples.

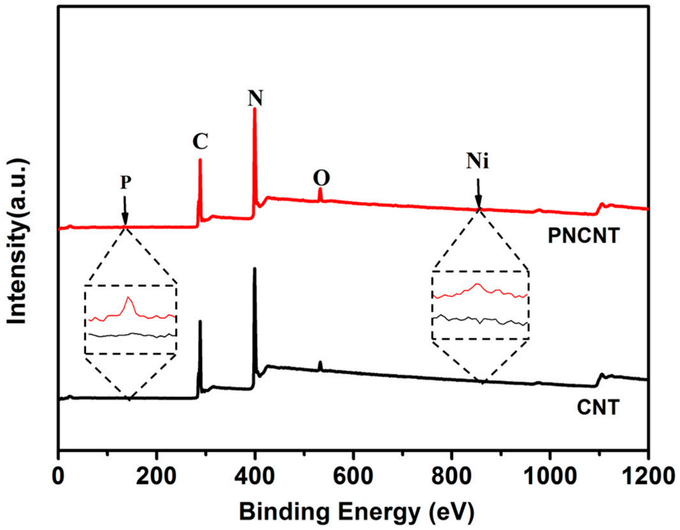

Figure S2. The XPS survey spectra of CNT and PNCNT samples.

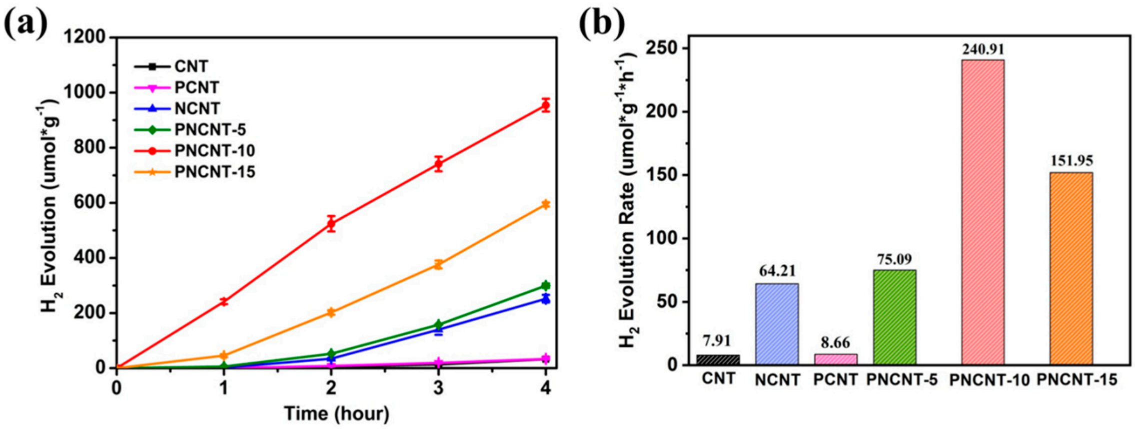

Figure S3. Time courses of photocatalytic H_2 evolution (a) and H_2 evolution rate (b) of CNT, NCNT, PCNT and PNCNT-x samples under visible light.

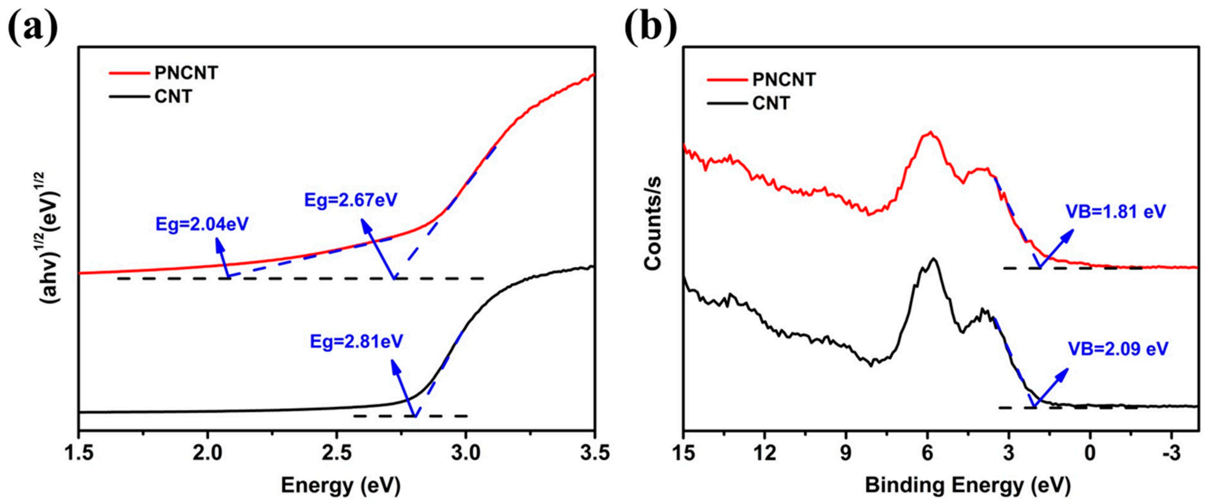

Figure S4. Plots of $(ahv)^{1/2}$ vs. photon energy (a) and XPS-VB of CNT and PNCNT (b).

Table S1.

The BET surface area, pore volume and average pore size of CNT, NCNT, PCNT and PNCNT samples.

Sample	A_{BET} (m²/g)	V_{pore} (cm³/g)	d_{pore}(nm)
CNT	4.1150	0.008975	1.8927
NCNT	8.7631	0.022687	1.7172
PCNT	8.7447	0.034028	3.0299
PNCNT	22.2571	0.093359	1.1247

Table S2.

Portion of each peak in the C 1s XPS spectra of CNT and PNCNT.

Sample	**Portion** (%)		
	C-C/C=C	C-NH_x	N-C=N
CNT	22.73	4.83	72.44
PNCNT	25.02	6.38	68.59

Table S3.

Comparison with other g-C_3N_4-baesd photocatalysts for HER rate.

Photocatalyst Composition	Sacrificial Agent	Cocatalysts	HER Rate (μ mol g^{-1} h^{-1})	Reference
CN/Ni-2	TEOA	N/A	86	[40]
MiL53(Fe)/g-C_3N_4	CH3OH	Pt	107.3	[41]
KTON/g-C_3N_4	TEOA	Au	186.5	[42]
CNT/Ni_2P-0.5%	TEOA	N/A	48.5	[43]
CNNT/CoP-2.45%	TEOA	N/A	153	[44]
Ag/g-C_3N_4	TEOA	N/A	18.97	[45]
PNCNT	TEOA	Pt	240.91	This work

Reference

40. Wu, G.; Liu, Q.; Ma, L.; Wu, H.; Li, Y.; Han, J.; Chen, G.; Xing, W. Strengthening reactive metal–support interaction to stabilize Ni species on the nitrogen vacancies of g-C_3N_4 for boosting photocatalytic H_2 production. *Catal. Sci. Technol.* **2021**, *11*, 7134-7140, doi:10.1039/d1cy01437d.
41. Pi, W.; Humayun, M.; Li, Y.; Yuan, Y.; Cao, J.; Ali, S.; Wang, M.; Li, H.; Khan, A.; Zheng, Z.; et al. Properly aligned band structures in B-TiO_2/MIL53(Fe)/g-C_3N_4 ternary nanocomposite can drastically improve its photocatalytic activity for H_2 evolution: Investigations based on the experimental results. *Int. J. Hydrogen Energy* **2021**, *46*, 21912-21923, doi:10.1016/j.ijhydene.2021.04.023.
42. Huang, X.; Zhang, X.; Mu, L.; Hu, M.; Dong, B.; Zhang, F. Synthesis of a novel nitrogen-doped $K_2Ti_6O_{13}$ nanorod with visible-light-driven water splitting performance promoted by fabrication of 1D/2D heterostructure. *Appl. Surf. Sci.* **2022**, *581*, doi:10.1016/j.apsusc.2021.152345.
43. Jiao, Y.; Li, Y.; Wang, J.; He, Z.; Li, Z. Double Z-scheme photocatalyst C_3N_4 nanotube/N-doped carbon dots/Ni_2P with enhanced visible-light photocatalytic activity for hydrogen generation. *Appl. Surf. Sci.* **2020**, *534*, doi:10.1016/j.apsusc.2020.147603.
44. Jiao, Y.; Li, Y.; Wang, J.; He, Z.; Li, Z. Novel B-N-Co surface bonding states constructed on hollow tubular boron doped g-C_3N_4/CoP for enhanced photocatalytic H_2 evolution. *J. Colloid Interface Sci.* **2021**, *595*, 69-77, doi:10.1016/j.jcis.2021.03.134.

45. Wu, Y.; Song, M.; Chai, Z.; Wang, X. Enhanced photocatalytic activity of $Ag/Ag_2Ta_4O_{11}/g\text{-}C_3N_4$ under wide-spectrum-light irradiation: H_2 evolution from water reduction without co-catalyst. *J. Colloid Interface Sci.* **2019**, *550*, 64-72, doi:10.1016/j.jcis.2019.04.087.
